# Imbalance between the function of Na^+^-K^+^-2Cl and K^+^-Cl impairs Cl^–^ homeostasis in human focal cortical dysplasia

**DOI:** 10.3389/fnmol.2022.954167

**Published:** 2022-10-17

**Authors:** Ru Liu, Yue Xing, Herui Zhang, Junling Wang, Huanling Lai, Lipeng Cheng, Donghong Li, Tao Yu, Xiaoming Yan, Cuiping Xu, Yueshan Piao, Linghui Zeng, Horace H. Loh, Guojun Zhang, Xiaofeng Yang

**Affiliations:** ^1^Guangzhou Laboratory, Guangzhou, China; ^2^Beijing Tiantan Hospital, Capital Medical University, Beijing, China; ^3^Beijing Institute of Brain Disorders, Capital Medical University, Beijing, China; ^4^Neuroelectrophysiological Laboratory, Xuanwu Hospital, Capital Medical University, Beijing, China; ^5^Department of Neurology, The Third Affiliated Hospital of Sun Yat-sen University, Guangzhou, Guangdong, China; ^6^Department of Functional Neurosurgery, Xuanwu Hospital, Capital Medical University, Beijing, China; ^7^Department of Pathology, Xuanwu Hospital, Capital Medical University, Beijing, China; ^8^Department of Pharmacology, Zhejiang University City College, Hangzhou, China

**Keywords:** epilepsy, focal cortical dysplasia, cation-chloride cotransporters, pyramidal neurons, seizure onset zone

## Abstract

**Objective:**

Altered expression patterns of Na^+^-K^+^-2Cl^–^ (NKCC1) and K^+^-Cl^–^ (KCC2) co-transporters have been implicated in the pathogenesis of epilepsy. Here, we assessed the effects of imbalanced NKCC1 and KCC2 on γ-aminobutyric acidergic (GABAergic) neurotransmission in certain brain regions involved in human focal cortical dysplasia (FCD).

**Materials and methods:**

We sought to map a micro-macro neuronal network to better understand the epileptogenesis mechanism. In patients with FCD, we resected cortical tissue from the seizure the onset zone (SOZ) and the non-seizure onset zone (non-SOZ) inside the epileptogenic zone (EZ). Additionally, we resected non-epileptic neocortical tissue from the patients with mesial temporal lobe epilepsy (MTLE) as control. All of tissues were analyzed using perforated patch recordings. NKCC1 and KCC2 co-transporters expression and distribution were analyzed by immunohistochemistry and western blotting.

**Results:**

Results revealed that depolarized GABAergic signals were observed in pyramidal neurons in the SOZ and non-SOZ groups compared with the control group. The total number of pyramidal neurons showing GABAergic spontaneous postsynaptic currents was 11/14, 7/17, and 0/12 in the SOZ, non-SOZ, and control groups, respectively. The depolarizing GABAergic response was significantly dampened by the specific NKCC1 inhibitor bumetanide (BUM). Patients with FCD exhibited higher expression and internalized distribution of KCC2, particularly in the SOZ group.

**Conclusion:**

Our results provide evidence of a potential neurocircuit underpinning SOZ epileptogenesis and non-SOZ seizure susceptibility. Imbalanced function of NKCC1 and KCC2 may affect chloride ion homeostasis in neurons and alter GABAergic inhibitory action, thereby contributing to epileptogenesis in FCDs. Maintaining chloride ion homeostasis in the neurons may represent a new avenue for the development of novel anti-seizure medications (ASMs).

## Introduction

Focal cortical dysplasia (FCD) is a common cause of refractory epilepsy (Blumcke et al., 2011). Histological features have shed light on the cytoarchitectural differences and underpinning developmental pathogenic mechanisms allowing for a more detailed categorization of FCD. However, from a functional perspective, the electrophysiological changes corresponding to these pathological changes and precise epileptogenic mechanisms of FCD remain unknown (Muhlebner et al., 2019).

Epilepsy results from an imbalance between excitatory and inhibitory synaptic transmission in the brain (Palma et al., 2017). Previous studies have documented decreased expression of γ-aminobutyric acid (GABA) receptor subunits, reduced γ-aminobutyric acidergic (GABAergic) interneuron, and decreased spontaneous postsynaptic GABA currents in pyramidal neurons. This may underpin the epileptogenic mechanisms of FCD (Talos et al., 2012; Zhou and Roper, 2014; Medici et al., 2016). FCD patients with epilepsy typically exhibit resistance to conventional anti-seizure medications (ASMs), especially GABA_A_ receptor agonists; this remains a clinical challenge. Recent studies have demonstrated that the Na^+^-K^+^-2Cl^–^ (NKCC1) and K^+^-Cl^–^ (KCC2) cotransporters alter GABAergic inhibitory function by regulating intracellular chloride concentration ([Cl^–^]_i_) (Doyon et al., 2016; Liu et al., 2019). Histological and molecular studies have revealed altered expression and distribution patterns of NKCC1 and KCC2 in the dysplastic neurons of patients with FCD exhibiting epilepsy (Aronica et al., 2007; Munakata et al., 2007; Sen et al., 2007). In addition, bumetanide (BUM), a specific blocker of NKCC1, has been reported to exhibit antiepileptic effects in several preclinical and clinical studies (Dzhala et al., 2005; Beleza, 2009; Eftekhari et al., 2013; Gharaylou et al., 2019; Ragot et al., 2021). This suggests that the expression and function changes in the chloride co-transporter play important roles in epileptogenesis. However, electrophysiological studies of the functional alterations underlying these changes remain limited. Cepeda et al. (2007) reported the depolarized equilibrium potential of GABA (E_GABA_) and prominent GABA_A_ receptor (GABA_A_R)-mediated spontaneous and evoked depolarizing responses in pediatric patients with severe FCD exhibiting epilepsy. Blauwblomme et al. (2019) demonstrated that the abnormal function and expression of the chloride transporter in neurons of patients with FCD exhibiting epilepsy may lead to the paradoxical depolarization of pyramidal neurons. However, these studies have inherent limitations, such as inevitable disputed [Cl^–^]_i_ caused by whole-cell recordings or the lack of intracellular recordings.

At the macroscopic level, based on electrocorticography (ECoG) data Rosenow and Luders (2001) defined the epileptogenic zone (EZ) as the area of the cortex necessary for the generation of recurrent seizures that needs to be removed to achieve seizure freedom. Recently, under diverse macroscopic diagnostic tools, different cortical zones have been conceptualized, such as the seizure onset zone (SOZ), which has been found to be characterized by anomalous EEG signals compared with the surrounding tissues (Jehi, 2018; Zijlmans et al., 2019). Although the temporal dynamics of epileptiform synchronization and epileptogenic networks are well described at the macroscopic level, the key features at the cellular and microcircuit levels cannot be captured by macroscopic evaluation. Therefore, little remains known about the microscopic characteristics and network changes of the EZ (Farrell et al., 2019). Clinicians were determined to gain a comprehensive understanding of epileptic mechanisms through multi-scale evaluations. Therefore, in this study, we selected the SOZ and non-seizure onset zone (non-SOZ) in the EZ of patients with FCD exhibiting epilepsy to assess disease-related changes at the cellular and microcircuit levels.

This study sought to compare the detailed electrophysiological characterization of GABAergic neurotransmission in pyramidal neurons in the SOZ and non-SOZ inside the EZ of patients with FCD with non-epileptic temporal neocortex and to correlate these features with the patterns of NKCC1 and KCC2. We probed whether depolarizing GABAergic action caused by the imbalanced function of NKCC1 and KCC2 cotransporters would perturb chloride ion homeostasis in neurons and contribute to the generation and propagation of epilepsy in patients with FCD.

## Materials and methods

### Patient selection and cortical tissue acquisition

In this study, 30 patients with intractable epilepsy were enrolled at Xuanwu Hospital of Capital Medical University from June 2018 to December 2019. Following a standardized preoperative evaluation, all patients underwent surgery to remove the EZ. Neocortical tissues were obtained from 20 patients with FCD and epilepsy. In addition, 10 patients with mesial temporal lobe epilepsy (MTLE) were chosen as the control group because of their relatively non-epileptic neocortical tissue, as suggested by previous studies (D’Antuono et al., 2004; Palma et al., 2006). The temporal neocortex of these patients with MTLE was characterized by no obvious structural aberration and minimal epileptic discharge based on intraoperative ECoG recording. The detailed clinical information and pathological characteristics of the enrolled patients are summarized in Table 1. Our research protocol was approved by the Medical Ethics Committee of Xuanwu Hospital, Capital Medical University. All patients were well informed about the study, and consents were obtained from them.

**TABLE 1:** Patients characteristics.

Patient/Sex/Age	Histology	Onset age; frequency	Sz types	ASMs, n; Name	EEG	MRI; PET	Surgery	Regions	Groups
**FCD**									
1/M/18.5 years	FCD Ia	15 years; 2–3 Sz/day	Focal impaired awareness seizure	2; OXC, LEV	Abnormal spikes in RT	Normal MRI; hypometabolism in RT	RT + RI cortectomy	RIT	SOZ
2/M/34 years	FCD Ia	7 years; NA	Focal impaired awareness seizure, GTCS	2; CBZ, VPA	Abnormal spikes in LT	Normal MRI; hypometabolism in LT	LT lesion- ectomy after SEEG	LT	SOZ non-SOZ
3/F/22.1 years	FCD IIa	10 years; NA	Focal aware/impaired awareness seizure	NA	Abnormal spikes in RF	Abnormal signals in RA; hypometabolism in RF and RT	RF cortectomy after SEEG	RMF	SOZ
4/F/28.8 years	FCD IIa	9 years; 1 Sz/several days	Focal aware/impaired awareness seizure, GTCS	2; LEV, LTG	Rhythmic posterior RMF spikes and waves	NA; hypometabolism in RF and RIT	RF and RT lesionectomy	RSF	SOZ
5/M/31 years	FCD IIa	2 years; 10 Sz/month	Focal impaired awareness seizure	2; OXC, VPA	Abnormal spikes in LF	Mild LH swelling; hypometabolism in LF, LT, and LP	LF, LT, LH lesionectomy after SEEG	LMF	SOZ
6/F/7 years	FCD IIb	4 years; 2 Sz/day	Focal aware seizure	2; OXC, LEV	Abnormal spikes in LF	NA	LF lesion-ectomy	LMF	SOZ
7/F/11.9 years	FCD IIIa	5 years; 1–2 Sz/week	Focal impaired awareness seizure	3; VPA, OXC, CBZ	Abnormal spikes in LT	LH abnormal signals; hypo-metabolism in LT, LP, LO	LATL after SEEG	LTP	SOZ
8/M/31.3 years	FCD IIIa	24 years; 3–5 Sz/month	AS	2; LTG, VPA	Rhythmic LT spikes and slow waves	Bilateral mild HS; hypometabolism in LT, LH	Mesial LT lobectomy after SEEG	LTP	SOZ
9/M/27.6 years	FCD IIIb + CG	9 years; 2 Sz/month	Focal impaired awareness seizure	2; CBZ, VPA	RP, RT slow waves	Encephalomalacia foci in RP; NA	RT + RP lesionectomy after SEEG	RIP	SOZ
10/M/11.1 years	CD	2 years; NA	Focal impaired awareness seizure, GTCS, SE	NA	Abnormal spikes in RF, RMT	R cerebral hemisphere malformation; NA	Lesion-ectomy + hemispherectomy	RT	SOZ
11/F/12 years	CD	2 years; >2 Sz/six months	Focal impaired awareness seizure, GTCS	2; OXC, LEV	Rhythmic RP, RO, RT, central gyrus spikes and waves	RSF, RP G/W matter blur-ring; hypometabolism in RSP, RIT	Lesionectomy	RT	SOZ
12/F/35 years	FCD Ia	20 years; 1–2 Sz/day	Focal impaired awareness seizure	1; CBZ	Rhythmic RF, RT spikes	Hypersignal in RH; hypo-metabolism in RT, RH	RATL after SEEG	RMT	non-SOZ
13/M/22.8 years	FCD Ib	11 years; >10 Sz/month	focal impaired awareness seizure	2; CBZ, PB	Abnormal spikes in RT	FLAIR hyper-signal in RH; hypometabo-lism in RT	RATL	RMT	non-SOZ
14/M/24.3 years	FCD IIa	10 years; 1–2 Sz/day	Focal impaired awareness seizure, GTCS	2; TPA, CBZ	RF spikes and waves	Normal MRI; hypometabo-lism in RF	RF cortectomy	RSF	non-SOZ
15/F/15 years	FCD IIb	5 years; 10–20 Sz/month	Focal impaired awareness seizure, GTCS	2; VPA, TPM	Rhythmic LF, LT spikes, and slow waves	FLAIR hypersignal in LF; NA	LF + LCC cortectomy after SEEG	LSF	non-SOZ
16/F/12 years	FCD IIb	5 years; 4 Sz/week	Focal impaired awareness seizure	2; OXC, LEV	Rhythmic RT spikes and slow waves	RT abnormal signals; NA	RT cortectomy after SEEG	RT	non-SOZ
17/F/11 years	FCD IIb	6 years; 1–2 Sz/week	Focal impaired awareness seizure	4; OXC, VPA, LTG, CLZ	Spikes and slow waves in LF, central area and LT	LACC G/W blurring; hypometabo-lism in LF and LP	LF + LCC cortectomy after SEEG	LSF	non-SOZ
18/M/26 years	FCD IIIb + CG	1 year; 1 Sz/month	Focal impaired awareness seizure	2; CBZ, VPA	Abnormal spikes in RF	Encephalomalacia in RF and RI	RF cortectomy after SEEG	RIF	non-SOZ
19/M/31.7 years	CD	29 years; 2–3 Sz/day	GTCS	1; CBZ	RT and RF slow waves	R cerebral hemisphere malformation; hypometa-bolism in RT, RH, and RIF	RF + RT cortectomy	RT	non-SOZ
20/F/34.1 years	CD	13 years; 4–5 Sz/week	Focal aware/impaired awareness seizure, GTCS	1; CBZ	Rhythmic RF and RT waves and spikes.	RHS and RF G/W blurring; hypometabo-lism in RF and posterior RIT	RF + RT cortectomy after SEEG	RIF	non- SOZ
**Con**									
21/F/30 years	HS	23 years; 1–2 Sz/day	Focal impaired awareness seizure	2;LEV, VPA	RT spike (or sharp) slow wave	RHS; hypo-metabolism in RT	RATL	RTP	Con
22/F/21 years	HS (WHO I)	1 year; 4–5 Sz/month	Focal impaired awareness seizure	4; LTP, PB, VPA, CBZ	LT spike (or sharp) slow wave	LHS; hypo-metabolism in LH	LATL	LTP	Con
23/F/32 years	HS (WHO I)	2 years; NA	Focal impaired awareness seizure GTCS	3; PB, LTG, CBZ	Rhythmic LT spikes	LHS; hypo-metabolism in LT and LH	LATL	LTP	Con
24/F/33 years	HS	26 years; NA	Focal impaired awareness seizure GTCS	2; OXC, TPM	Rhythmic RH spikes	RHS; hypo-metabolism in RH	RATL	RTP	Con
25/M/48.5 years	HS (WHO I)	30 years; 1–3 Sz/day	Focal impaired awareness seizure GTCS	NA	LT slow waves and spikes	LT abnormal signals; NA	LATL	LTP	Con
26/M/45.7 years	GG	18 years; >3 Sz/week	Focal impaired awareness seizure, GTCS	2; CBZ, LEV	Rhythmic RT spikes	Hypersignal in RH; hypometabolism in RMT	RATL	RTP	Con
27/M/27 years	HGM	16 years; 1–2 Sz/week	Focal impaired awareness seizure	3; TPM, VPA, CBZ	Rhythmic RT spikes and slow waves	Normal MRI; hypometabolism in RP and RT	RATL	RTP	Con
28/M/39.3 years	HGM	24 years; 1–2 Sz/day	Focal impaired awareness seizure, GTCS	1;OXC	Abnormal spikes in LT	Bilateral mild HS; NA	LATL	LTP	Con
29/F/38 years	HS	23 years; 3–4 Sz/day	Focal aware/impaired awareness seizure	1;CBZ	Abnormal spikes in LT	Reduced LT; hypometabolism in LP, LF, LT	LATL	LT	Con
30/M/6 years	CG	4 years; 3–4 Sz/day	Focal aware/impaired awareness seizure	1;VPA	Abnormal spikes in LT	Reduced volume of LH; NA	LATL	LT	Con

ACC, anterior cingulate; ASM, antiseizure medicine; AS, atypical seizure; ATL, anterior temporal lobectomy; CBZ, carbamazepine; CG, cicatricial gyrus; CLZ, clonazepam; EEG, electroencephalography; F, female; FCD, focal cortical dysplasia; F, frontal lobe; GTCS, generalized tonic-clonic seizures; GG, ganglioglioma; G/W, gray/white; H, hippocampus; HGM: heterotopic gray matter; HS, hippocampal sclerosis; I, insular; L, left; LCM, lacosamide; LEV, levetiracetam; IF, inferior frontal gyrus; LRZ, lorazepam; LTG, lamotrigine; M, male; MF, middle frontal lobe; MT, middle temporal gyrus; NA, information not available; O, occipital lobe; OXC, oxcarbazepine; PB, Phenobarbital; PHT, phenytoin; RA, right amygdaloid nucleus; PPA, para-hippocampal place area; RP, right parietal lobe; RT, right temporal lobe; SE, status epilepticus; SF, superior frontal gyrus; Sz, seizure(s); T, temporal lobe; TP, temporal pole; TPM, topiramate; VPA, valproic acid.

**FIGURE 1:**
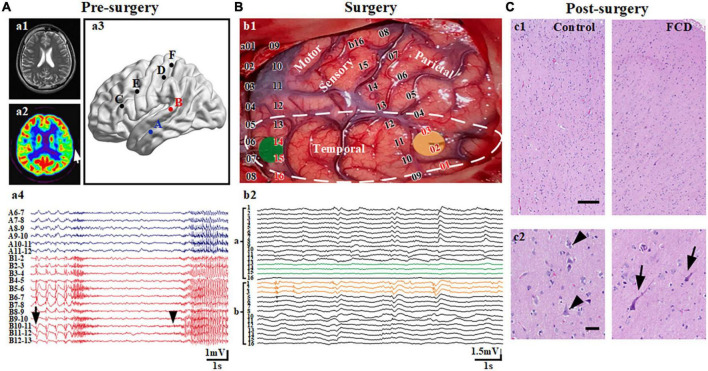
Localization of the seizure onset zone (SOZ) and non-seizure onset zone (non-SOZ). **(A)** Presurgical evaluation and confirmation of SOZ. **(a1,2)** Represents an area of hypometabolism in the left middle temporal lobe (arrow) in a PET scan of the patient with focal cortical dysplasia (FCD) having a negative MRI. **(a3,4)** Represents spontaneous recurrent rhythmic interictal spikes (arrow) and ictal discharges (arrowhead) restricted to the region in the left middle temporal gyrus from electrode B in the stereo-electroencephalograph recordings. **(B)** Reconfirmation of neocortical samples during surgery. **(b1)** The yellow and green areas reflect the areas of tissue samples used for electrophysiological recording. **(b2)** Recordings based on electrocorticography (ECoG) showed continuous epileptiform discharges (yellow line) and normal background activity (green line) in the temporal region. The area surrounded by a white dotted line is the region being removed. **(C)** Post-surgery histological examination with FCD IIa. **(c1)** Hematoxylin and eosin (H and E)-stained section of brain tissues of control and FCD groups. **(c2)** The left section shows relative normal shaped and arranged neurons (arrowheads) in the lateral neocortex from patients with mesial temporal lobe epilepsy. The right section represents the extension of the area in the first panel showing dysmorphic neurons with disrupted cortical lamination (arrows). Scale bars represent 500 and 100 μm for the first and second panels, respectively.

Each patient recruited in the study underwent standardized preoperative evaluation procedures to delineate the EZ and locate the SOZ, including detailed clinical history, neurological examinations, video-electroencephalographic recordings (vEEG), neuropsychological examinations, and neuroimaging studies, etc. Neuroimaging studies include high-resolution magnetic resonance imaging (MRI, 3T), magnetoencephalography, and 18-fluorodeoxyglucose positron emission tomography (FDG-PET). As mentioned above, Rosenow and Luders (2001) defined the EZ as the area of cortex that is indispensable for the generation of epileptic seizures. An integral component for the delineation of the EZ is the seizure onset zone (SOZ): the area of cortex that initiates clinical seizures as determined predominantly by intracranial investigations. Inside the EZ, two blocks of neocortical tissue (1 cm × 1 cm × 1 cm) were defined as SOZ and non-SOZ. However, there is currently no clear definition of “non-SOZ.” In this study, SOZ is confirmed as the area of cortex that initiates clinical seizures by combining presurgical intracranial investigations and symptomatology (Figure 1A, electrode B). For the other area expect SOZ in the surgical resection, as our previous study, we choose “non-SOZ” as one of brain tissue which is also located in the area to be excised but showing the least abnormal electrophysiological recordings under subdural recording (Figure 1B; Cheng et al., 2022).

Given that the brain tissue is taken during surgery, the surgeon cannot cut off the blood supply in order to keep the brain tissue alive before collecting the brain tissue. When we collected the first two samples (SOZ and non-SOZ) from the first patient, it was shown that collecting two brain tissues at the same time would cause more serious bleeding which did inevitable damages to the patient. Therefore, from the second patient, we adopted a double-blind and random sampling method, and each patient randomly collected one piece of brain tissue. All patients with FCD were confirmed by a postoperative histological examination (Figure 1C). In summary, we divided our experiment into three groups: the SOZ, non-SOZ, and control groups.

### Brain tissue slice preparation

After being obtained in the operating room, neocortical tissues were immediately placed into an ice-cooled and oxygenated (95% O_2_ and 5% CO_2_) cutting solution containing 220 mM sucrose, 2.5 mM KCl, 1.25 mM NaH_2_PO_4_, 25 mM NaHCO_3_, 0.5 mM CaCl_2_, 7 mM MgCl_2_, and 10 mM D-glucose (pH 7.4; osmolarity maintained at 340 mOsm/kg), and transferred to our electrophysiological laboratory within 5–10 min. Subsequently, meninges and blood clots were gently removed, and 400 μm neocortical transverse slices were cut using a vibratome (VT 1000S; Leica Microsystems, Wetzlar, Germany) and incubated in oxygenated (95% O_2_ and 5% CO_2_) artificial cerebrospinal fluid (ACSF) containing (in mM) 125 NaCl, 2.5 KCl, 1.25 NaH_2_PO_4_, 26 NaHCO_3_, 2 CaCl_2_, 2 MgCl_2_, and 10 D-glucose (pH 7.3; osmolarity maintained at 300–310 mOsm/kg) at room temperature for at least 1 h before recording.

### Perforated patch-clamp recording and data analysis

**FIGURE 2:**
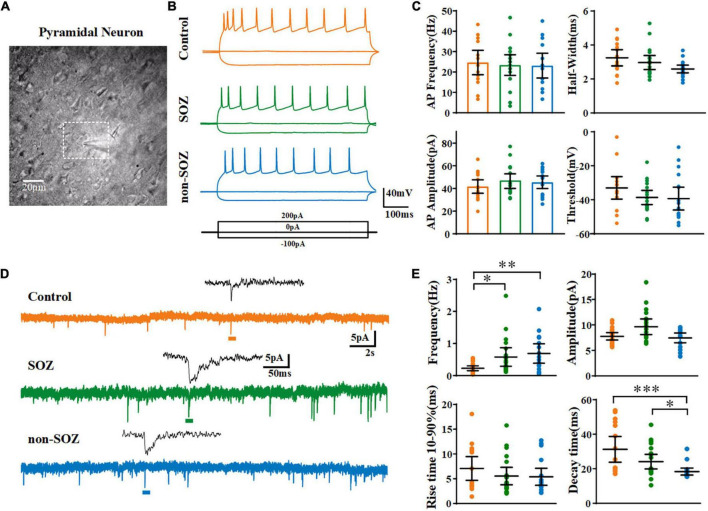
Electrophysiological characteristics and spontaneous excitatory postsynaptic currents of pyramidal neurons (PN) among groups. **(A)** Photomicrographs of individual PN within the dysplastic cortex. **(B)** Representative traces and action potential firing of pyramidal neurons from the SOZ, non-SOZ, and control groups with –100, 0, 200 pA injection at the resting membrane potential. **(C)** Representative traces of spontaneous excitatory postsynaptic currents obtained in the three groups. **(D)** Comparison of the electrophysiological characteristics of pyramidal neurons across the three groups *N* = 19, 18, 16 for SOZ, non-SOZ, and control groups, respectively. **(E)** Comparison of spontaneous excitatory postsynaptic currents of pyramidal neurons across the three groups. *N* = 20, 18, 16 for SOZ, non-SOZ, and control groups, respectively. *p < 0.05, ^**^p < 0.01, ^***^p < 0.01. Data was shown as mean ± SEM.

Following incubation, the brain slices were submerged in a recording chamber perfused with oxygenated ACSF at a rate of 2–2.5 mL/min at room temperature. Perforated patch-clamp recordings were performed on pyramidal neurons in the neocortex and viewed under an infrared differential interference contrast microscope with a Nikon 40 water immersion lens (ECLIPSE FN1, Nikon Corp., Tokyo, Japan). Recordings were performed in layers III and IV in the non-SOZ and control groups. In the SOZ group, in which the lamina was not clearly defined, layers III and IV were generally located according to the non-SOZ lamina in the middle region of the dysplastic cortex (Figure 2A). Pyramidal neurons were identified based on their triangular-shaped cell bodies and relatively simple axons and were confirmed by electrophysiological recordings as displaying longer action potential (AP) half-widths and lower peak AP frequency (Avermann et al., 2012). Recording electrodes (resistance, 4–6 MΩ) were prepared from borosilicate glass capillaries (B15014F, Vital Sense Scientific Instruments Co., Ltd., Wuhan, China) using a horizontal pipette puller (P-1000 Next Generation Micropipette Puller, Sutter Instrument, CA, USA). The pipette solution contained (in mM) 135 KCl, 10 HEPES, 0.5 CaCl_2_, 2 MgCl_2_, and 5 EGTA (pH 7.3 adjusted with KOH; osmolarity maintained at 290–300 mOsm/kg). Gramicidin was stocked at an initial concentration of 16 mg/ml and then diluted with pipette solution to reach a final concentration of 80 μg/ml. The electrode tip and barrel were backfilled with pipette solution and gramicidin-containing pipette solution, respectively. Following the formation of a tight seal and after the series resistance (Rs) decreased and stabilized at 30–110 MΩ, we started recording. Recordings were ended when a rapid decrease in Rs and a “leak-like” current were observed, indicating that the cell had entered the conventional whole-cell configuration by rupture of the membrane seal. The resistance and capacitance of the pipette have been compensated. Liquid junction potential was not corrected.

We first recorded the resting membrane potential (V_m_), membrane conductance, and firing properties to identify pyramidal neurons in the current-clamp mode. Next, we recorded spontaneous excitatory postsynaptic currents (sEPSCs) for 3 min at a holding potential of −70 mV in the voltage-clamp mode. Subsequently, we acquired pharmacologically isolated GABAergic sPSCs by applying 6-cyano-7-nitroquinoxaline-2,3-dione (CNQX, 10 μM) and DL-2-amino-5-phosphonopentanoic acid (DL-AP5, 100 μM) to block α-amino-3-hydroxy-5-methylisoxazole-4-propionic acid (AMPA) and *N*-methyl-D-aspartate (NMDA) receptors. BUM (10 μM) was then added to assess its effects on GABAergic sPSCs. The sEPSCs were automatically detected at amplitude adjusted above the root 2 mean square noise level using Clampfit 10.5 (Molecular Devices, CA, USA). Cumulative amplitudes were calculated by summating amplitudes of all events within 180 s periods.

In this study, to probe the E_GABA_ of pyramidal neurons, we first determined the neuronal firing patterns in response to depolarizing current injections prior to perfusion with tetrodotoxin. We applied GABA in the chamber with a pulsed fashion immediately during the ramp test. A hand-made fast drug delivery systems was used to apply GABA (100 μM, containing: CGP 35348, 1 μM; TTX, 1 μM) from a pipette resting 10 –20 μm above the slice at the position of the recorded soma. The system enabled quick fluid changes with a speed of 1 ml/min and a diameter of about 20 μm for its tip. Voltage ramps −80 to + 10 mV over 100 ms applied from a holding potential of −70 mV in the absence and presence of GABA were used to determine E_GABA_. The membrane voltage at which the current traces, obtained in the presence and absence of GABA, crossed was measured as the apparent E_GABA_. Then, voltage ramps were repeated following the addition of BUM (10 μM). The membrane voltage at which the current traces, obtained in the presence and absence of BUM, crossed was measured as the apparent E_*BUM*_. In addition, the driving force for GABA production (DF_GABA_ = E_GABA_–Vm) was measured.

### Immunohistochemistry

The brain tissue was fixed in 10% formalin and embedded in paraffin. Paraffin-embedded tissues were sectioned at 6 μm and heated in a microwave oven (60°C for 60 min). The sections were thereafter deparaffinized in xylene and rehydrated sequentially in graded concentrations of alcohol. Sections from the SOZ, non-SOZ, and control groups were probed with an anti-KCC2 antibody (Millipore, #07-432, Boston, MA, USA, 1:200) overnight at 4°C. The slides were then incubated with horseradish peroxidase-conjugated secondary antibodies at room temperature for 1 h. Immunolabeling was visualized using the avidin-biotin conjugation method and 3,3′-diaminobenzidine. The sections were next counterstained with hematoxylin.

### Immunofluorescence staining

Brain slices were post-fixed with 10% formalin, washed three times with PBS, incubated for 30 min with 0.3% Triton, and then blocked with 1% goat serum for 1 h to avoid binding of non-specific antibodies. Sections were then incubated at 4°C overnight with the following primary antibodies: anti-NKCC1 (Millipore, PA5-118800, Boston, MA, USA, 1:200), anti-NeuN (Sigma-Aldrich, St. Louis, MO, USA, MAB377, 1:200). Secondary antibodies conjugated to Alexa Fluor 488 or 594 (1:1,000; Invitrogen) were applied for 1.5 h at room temperature. Slides were mounted with Fluoroshield containing DAPI (Ab104139; Abcam) and observed with a confocal microscope (LSM710; Zeiss, Oberkochen, Germany).

### Western blot analysis

Total protein was quantified using a BCA Protein Assay Kit (Solarbio, Beijing, China). Samples were electrophoresed on 5 and 10% Tris–HCl gels, transferred onto polyvinylidene difluoride membranes, and incubated with primary antibodies (anti-KCC2, Millipore, 1:1,000). GAPDH (mouse monoclonal IgG, CST, Danvers, MO, USA 1:5,000) was used as the reference protein. Horseradish peroxidase-conjugated secondary antibodies (anti-rabbit IgG and anti-mouse IgG; LI-COR, USA) were used to bind the primary antibodies. The bands were visualized using a two-channel infrared (IR) scanner (Odyssey, NE, USA). Densitometric analysis of the protein signals was carried out using the ImageJ software (version 6.0; NIH, Bethesda, MD, USA). The target protein levels were normalized to internal control.

### Statistical analysis

All data distributions were assessed with the Shapiro–Wilk test. For the normal distribution, data were analyzed using Student’s *t*-test or one-way ANOVA with *post-hoc* tests. Results were expressed as mean ± standard error of the mean (SEM), with significance set at *p* < 0.05. For the skewed distribution, analysis was carried out using the non-parametric Kruskal–Wallis test followed by Dunn’s multiple comparisons or Mann–Whitney *U*-test. Results were expressed as medians and interquartile range (IQR), with significance set at *p* < 0.05.

## Results

### Clinical information

First, we analyzed the clinical characteristics of the patients included in this study. No significant differences in the mean duration of epilepsy were observed across the groups. Briefly, the male/female ratio of patients with FCD exhibiting epilepsy enrolled in the study was 1:1, the mean duration of epilepsy being 13.5 ± 1.8 years and the average age at surgery being 22.9 ± 2.1 years old. The EZ in the FCD was localized in frontal (*n* = 9), temporal (*n* = 10), or posterior quadrants (*n* = 1).

### Comparison of electrophysiological properties in pyramidal neurons among seizure the onset zone, non-seizure the onset zone, and control groups

Using perforated patch-clamp recording, we first compared the basic electrophysiological properties of pyramidal neurons across the three groups in the current-clamp mode. No significant differences were observed across the groups in terms of average V_m_, cell capacitance (C_m_), input resistance (R_N_), or membrane time constant (Table 2). No significant differences were observed across the pyramidal neurons of the three groups regarding the characteristics of the action potentials induced by the injection of a + 300 pA current (Figures 2B,C and Supplementary Figure 1).

**TABLE 2:** Comparison of the intrinsic membrane properties of pyramidal neurons among different groups.

	FCD (SOZ, *N* = 20)	FCD (non-SOZ, *N* = 18)	Control (*N* = 16)
RMP (mV)	−74.4 (−64.1 to −78.3)	−76.5 (−72.0 to −79.8)	−73.1 (−69.9 to −78.1)
Cm (pF)	109.3 (81.2 to 129.9)	88.7 (83.25 to 104.8)	99.1 (77.2 to 119.2)
R_N_ (MΩ)	226.2 ± 26.4	189.4 ± 20.9	229.9 ± 22.0
Time constant (ms)	5.0 ± 0.5	4.2 ± 0.5	5.0 ± 0.4

RMP, resting membrane potential; Cm, membrane capacitance; R_N_, input resistance. Values are reported as mean ± SEM; medians and interquartile range (IQR), with significance set at *p* < 0.05. There was no significant difference among three groups in the above parameters.

Next, we recorded the sEPSCs of pyramidal neurons in the SOZ, non-SOZ, and control groups held at −70 mV. The average frequency of sEPSCs on pyramidal neurons was significantly lower in the control group than in the SOZ group (median = 0.25 Hz, IQR = 0.1–0.28 Hz, *n* = 17 vs. median = 0.36 Hz, IQR = 0.22–0.85 Hz, *n* = 20, p = 0.02) and non-SOZ group (median = 0.53 Hz, IQR = 0.28–1.05 Hz, *n* = 16, *p* = 0.003). However, no significant difference was observed between the SOZ and non-SOZ groups. The median amplitude of sEPSCs on pyramidal neurons was not significantly different among the three groups. The decay time was smaller in the non-SOZ group (median = 17.02 ms, IQR = 15.95–18.54 ms) than in the control group (median = 23.11 ms, IQR = 19.61–47.29 ms, *p* = 0.0001) and the SOZ group (median = 19.73 ms, IQR = 18.62–31.47 ms, *p* = 0.02). In addition, no significant differences in the sEPSC rise time were observed across the groups (Figures 2D,E).

**FIGURE 3:**
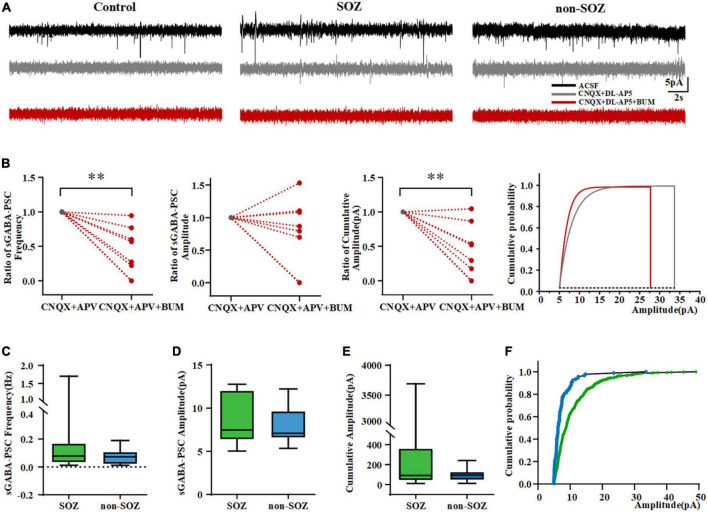
Pyramidal neurons in the SOZ and non-SOZ of focal cortical dysplasia (FCD) showing depolarizing bumetanide-sensitive GABA_A_ receptor responses. **(A)** Altered inhibitory GABAergic signaling in SOZ and non-SOZ groups. **(B)** Bumetanide significantly dampened the frequency and cumulative amplitude of GABA_A_R-mediated sPSCs in FCD patients *N* = 10. **(C–E)** GABA_A_R-mediated sPSCs are shown in the SOZ and non-SOZ groups. **(F)** Cumulative GABA_A_R-mediated sPSCs amplitude probability distributions for pyramidal neurons from SOZ and non-SOZ groups. ^**^p < 0.01. Data was shown as medians and interquartile range (IQR).

In a previous study, Talos et al. applied glutamatergic receptor antagonists to isolate and confirm the GABAergic components (GABA_A_ receptor-mediated spontaneous postsynaptic currents, GABA_A_R-mediated sPSCs) with picrotoxin at a holding potential close to RMP in patients with tuberous sclerosis (Talos et al., 2012). Based on their study’s results, we assessed the effects of GABAergic neurotransmission on sPSCs after blocking glutamatergic receptors to investigate the alterations in GABAergic neurotransmission in human dysplastic epileptic tissues. We first recorded sEPSCs in pyramidal neurons under normal ACSF perfusion in the SOZ, non-SOZ, and control groups, followed by the complete blockade of AMPA and NMDA receptors with CNQX and DL-AP5. Consistent with a previous study, under conditions of complete AMPA and NMDA receptor blockade, residual GABA_A_R-mediated sPSCs disappeared completely in the control group. However, GABA_A_R-mediated sPSCs remained present in the SOZ and non-SOZ groups, although they were significantly reduced (Figure 3A).

We assessed the effects of BUM, a NKCC1 blocker, on GABA_A_R-mediated sPSCs. We first probed how BUM would affect sPSCs by regulating neuronal action potentials. Results demonstrated that BUM did not change the frequency, peak amplitude, threshold, or half-width of the neuronal action potential (data not shown). We normalized GABA_A_R-mediated sPSCs before and after the application of BUM (10 μM). BUM reduced GABA_A_R-mediated sPSCs frequency (from 1 to 0.25; *n* = 10, *p* = 0.002) and cumulative amplitude (from 1 to 0.24, *n* = 10, *p* = 0.004) in FCD patients (Figure 3B). No significant effect of BUM was observed on GABA_A_R-mediated sPSCs amplitude (Figure 3B).

### Features of γ-aminobutyric acidergic transmission

We found no GABA_A_R-mediated sPSCs on pyramidal neurons in the control group. However, GABA_A_R-mediated sPSCs were more common in neurons in the SOZ group (11/18, 61.11%) than in the non-SOZ (7/18, 38.89%). There was no significant difference in the frequency of GABA_A_R-mediated sPSCs (median = 0.08 Hz, IQR = 0.03–0.17 Hz, *n* = 11 vs. median = 0.07 Hz, IQR = 0.02–0.11 Hz, *n* = 7, *p* > 0.05), the amplitude of GABA_A_R-mediated sPSCs (median = 7.5 pA, IQR = 6.4–12.0 pA, *n* = 11 vs. median = 7.1 pA, IQR = 6.6–9.6 pA, *n* = 7, *p* > 0.05) and the cumulative amplitude of GABA_A_R-mediated sPSCs (median = 90.7 pA, IQR = 44.7–360.2 pA, *n* = 11 vs. median = 95.0 pA, IQR = 48.9–125.4 pA, *n* = 7, Mann–Whitney *U-*test, *p* > 0.05) in the pyramidal neurons of between SOZ and non-SOZ groups (Figures 3C–E). Cumulative GABA_A_R-mediated sPSCs amplitude distribution from cells depicted in SOZ showed was significantly higher in SOZ compared with non-SOZ group (*P* < 0.001, Mann–Whitney *U-*test, Figure 3F).

### More depolarized E_GABA_ in seizure the onset zone of focal cortical dysplasia

**FIGURE 4:**
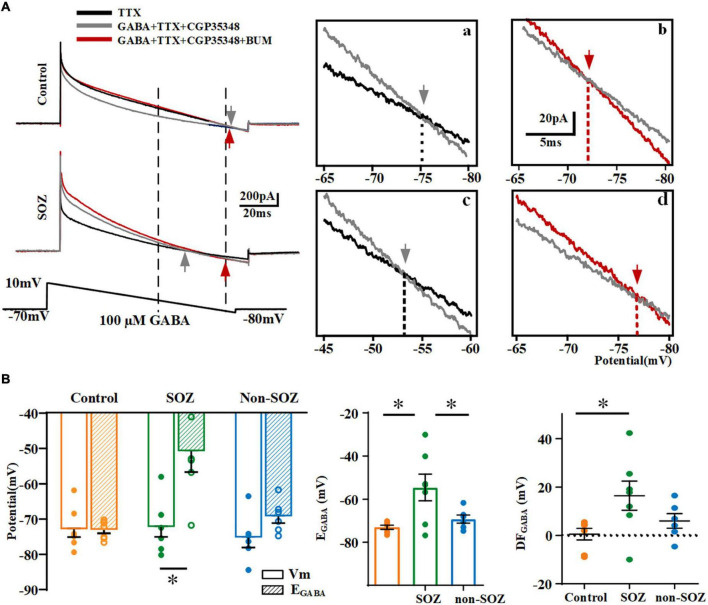
Comparison of reversal potentials of GABA (E_GABA_) across groups. **(A)** Gramicidin-perforated-patch recordings exhibiting current traces of pyramidal neurons from SOZ **(a)** and control groups **(b)** during hyperpolarizing ramps (bottom) in the absence of GABA (black line), with the application of 100 μM GABA (gray line), and with the application of 100 μM GABA and 10 μM BUM (red line). The ramp potential at which the current traces without GABA and with GABA, without BUM and with BUM intersected, represent E_GABA_ (gray arrow in inset) and E_BUM_ (red arrow in inset). Vertical dashed lines intersecting the command potential schematic show the range of command voltage in the boxed area. Insets: expanded traces of boxed regions illustrate the E_GABA_ (gray arrow) and E_BUM_ (red arrow) of a pyramidal neuron in SOZ **(a,c)** and control groups **(b,d)**. **(B)** Summary plot of E_GABA_ and DF_GABA_ across groups. *p < 0.05. Data was shown as mean ± SEM.

E_GABA_ reflects the potential at which currents are generated during the voltage ramp in the absence and presence of GABA intersect (Figure 4A). The average E_GABA_ for each neuron measured in the different groups is represented in Figure 4B. The E_GABA_ on pyramidal neurons in the SOZ group (−54.5 ± 6.2 mV, *n* = 7) was significantly shifted to more positive values compared to that in the control group (−73.0 ± 1.0 mV, *n* = 7, *p* < 0.05) and non-SOZ group (−69.2 ± 1.9 mV, *n* = 6, *p* < 0.05, Figure 4B). The polarity of GABAergic response is affected by the driving force of GABA. E_GABA_ (−54.5 ± 6.2 mV, *n* = 7) was more positive than RMP (71.6 ± 2.5 mV, *n* = 7, Student’s *t*-test, *p* < 0.05) in the SOZ group. In contrast, no significant differences were observed between E_GABA_ and RMP in the non-SOZ (−75.2 ± 2.8 mV, *n* = 6 vs. −69.2 ± 1.9 mV, *n* = 6) and control groups (−73.6 ± 2.4 mV, *n* = 7 vs. −73.0 ± 1.0 mV, *n* = 7). DF_GABA_ was significantly higher in the SOZ group than in the control group (median = 17.85 mV, IQR = 8.43–15.55 mV, *n* = 7 vs. median = 4.29 mV, IQR = −8.18-4.617 mV, *n* = 7, *p* > 0.05 Figure 4B). In contrast, no significant difference was observed in the DF_GABA_ of the non-SOZ group compared to the control group. Application of BUM did not alter E_GABA_ in the control group (−75.1 vs. −71.2 mV), but E_GABA_ shifted to more negative values in the SOZ group (−52.9 vs. −75.0 mV) (Figure 4A).

### Expression and distribution of K^+^-Cl^–^ in human epileptic focal cortical dysplasia and non-epileptic cortical tissues

**FIGURE 5:**
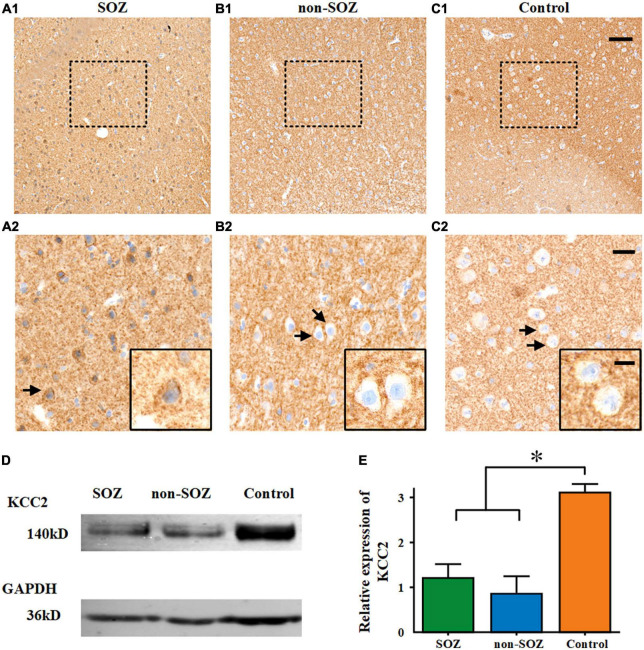
Comparison of KCC2 expression across all three groups. A diaminobenzidine reaction product was used for the immunohistochemical detection of KCC2 using a hematoxylin counterstain. **(A–C)** Sub-cellular KCC2 distribution in the three groups. High peri-somatic KCC2 localization was assessed in the SOZ of patients with FCD and epilepsy. Cytoplasmic expression was absent in the non-SOZ of the same patient and the control group. **(A2–C2)** Represent magnified images of the enclosed areas in **(A1–C1)**. Black frames in **(A2–C2)** depict different sub-cellular KCC2 distribution (arrows). Scale bars represent 100 μm for **(A1–C1)**, 30 μm for **(A2–C2)**, and 10 μm for the black frame in **(A2–C2)**. **(D,E)** Expression in total homogenates from specimens in SOZ, non-SOZ, and control groups. Expression of GAPDH (as a reference protein) as seen in the same protein extracts. *p < 0.05. Data was shown as mean ± SEM.

In addition to the electrophysiological results indicated significant depolarizing GABAergic signaling in neurons in the SOZ group, we assessed the neuronal expression of NKCC1 and KCC2 in the three groups (Figure 5). As previous studies, NKCC1 showed a wide distribution in a variety of tissues and cell types within and outside the nervous system (Virtanen et al., 2020). In our study, we also tried to explore the subcellular distribution of NKCC1 using immunofluorescence but found that no significant differences in the subcellular location of NKCC1 were observed across the groups. Strong NKCC1 immunoreactivity was detected in most NeuN-expressing normal-sized neurons in all the groups. Immunostaining for NKCC1 was present in the soma and dense intra-somatic staining (Supplementary Figure 2).

Diffuse neuropil staining for KCC2 was observed in the FCD cortical tissue as well as in the control temporal neocortex. The neurons in the SOZ showed that KCC2 was internalized into the cells and that its distribution on the cell membrane was significantly reduced. However, there was no obvious KCC2 cell internalization in the non-SOZ and control groups. Histological analyses revealed cytoplasmic KCC2 accumulation in neurons in the SOZ group, whereas cytoplasmic KCC2 staining was absent in the non-SOZ and control groups (Figures 5A–C). A western blot analysis showed that the expression of KCC2 protein in the SOZ and non-SOZ groups was significantly decreased compared with that in the control group (Figures 5D,E).

## Discussion

In this study, we assessed the electrophysiological characteristics and depolarizing synaptic activity of pyramidal neurons in patients with FCD and epilepsy. In particular, we probed the effects of imbalanced function of NKCC1 and KCC2 on GABAergic signaling in pyramidal neurons inside the SOZ, non-SOZ within the EZ of the patients with FCD, and non-epileptic neocortex in patients with MTLE (control). We sought to establish a relationship between macroscopic epileptogenesis and microscopic cell-to-cell communication to better understand the epileptogenic network of FCD through a micro-macro neuro-electrophysiology lens. We observed depolarizing GABAergic signaling in pyramidal neurons located in the SOZ and non-SOZ of the FCD, but not in the control group. Although there is no significant difference between SOZ and non-SOZ in terms of frequency and intensity of depolarizing GABAergic responses at this microscopic level, however, there were more neurons with depolarizing GABAergic responses and higher E_GABA_ in the SOZ than in the non-SOZ, which may explain the macroscopic differences between the SOZ and non-SOZ in patients with FCD. In addition, we revealed that depolarizing GABAergic neurotransmission in FCD patients, which can be reversed by BUM, is related to the imbalanced function of NKCC1 and KCC2 in the SOZ. This is the first comparative study of depolarizing GABAergic signals in the SOZ, non-SOZ, and non-epileptic cortical regions. Our findings provide important evidence of a potential neurocircuit underlying SOZ epileptogenesis and non-SOZ seizure susceptibility. These results also point to a possible mechanism for epileptogenesis in FCD at the cellular and microcircuit levels, highlighting a novel therapeutic avenue for patients with FCD and epilepsy.x

### Establishing micro-macro electrophysiological recordings to gain a better understanding of the epileptogenic network of patients with focal cortical dysplasia exhibiting epilepsy

Accurate delineation of the EZ is critical to determining which patients with refractory epilepsy are appropriate candidates for surgical intervention (Feindel et al., 2009). Although the EZ remains a theoretical concept, five cortical zones (including the SOZ) related to the EZ are roughly defined in presurgical evaluations (Jehi, 2018; Zijlmans et al., 2019). The precise definition of the SOZ has long been thought to accurately delineate the EZ, but it has been found that the EZ is often more extensive than the SOZ, and the precise boundary of the EZ is poorly defined (Rosenow and Luders, 2001). Rasmussen et al. proposed that removing the SOZ was necessary but insufficient to achieve lasting seizure-free survival (Rasmussen, 1983), which indicates the potential involvement of the non-SOZ within the EZ in epileptogenesis. Currently, epilepsy is considered to be a disease of neural networks (Gonzalez Otarula et al., 2019). Successful surgical outcomes depend on the analysis of seizure-generating networks. For a network-based analysis, focusing on regions in the EZ beyond the SOZ to understand the mechanisms of epileptogenesis is crucial (Badier et al., 2015; Paz and Huguenard, 2015; Zijlmans et al., 2019). In addition, a well-recognized, major clinical problem is that most studies locating the SOZ and EZ mainly emphasize the macroscopic level, with little concern over the microscopic changes involved. Given the markedly different EEG signals between the SOZ and non-SOZ in patients with FCD, we assessed the underlying neurotransmission patterns, particularly the depolarizing GABAergic signals, to uncover potential microscopic distinctions that contribute to the macroscopic differences.

Another challenge in human clinical research remains the acquisition of healthy, non-epileptic brain tissue, which would otherwise not warrant surgical resection in a clinical setting. Here, we chose the temporal neocortex from patients with MTLE as our control group for the following reasons: (1) temporal neocortical tissue lies far from the EZ and is not characterized by any obvious structural aberration (D’Antuono et al., 2004); (2) temporal neocortical tissue has been shown to have the least abnormal transcription of Cl^–^ transporters and highest E_GABA_ compared to the subiculum and hippocampal proper in patients with MTLE, which indicates relatively balanced NKCC1 and KCC2 level (Palma et al., 2006); and (3) temporal neocortical tissue is often resected during operations for such patients with epilepsy.

### GABA_A_ receptor-mediated depolarization caused by imbalanced function of Na^+^-K^+^-2Cl and K^+^-Cl^–^ promotes epileptogenesis in patients with focal cortical dysplasia exhibiting epilepsy

The recruitment of glutamatergic neurons is a fundamental component of seizures (Farrell et al., 2019). As 80% of neocortical neurons are excitatory glutamatergic pyramidal neurons (with a higher percentage in CD) (Deukmedjian et al., 2004), pyramidal neurons in FCD are maintained in an immature state with hyperexcitable properties in the malformed cortex (George and Jacobs, 2011). Pathologically interconnected neurons have been hypothesized to gather as novel pathological microdomains, leading to interacting networks that generate hypersynchronous discharges that trigger a seizure (Bragin et al., 2000). Previous reports have indicated that the key features of microcircuits captured by microscopic recordings in different brain regions associated with the EZ are significantly different (Stead et al., 2010; Blauwblomme et al., 2019). The heterogeneity of these microcircuits plays an important role in the formation of the macro-networks. Our results demonstrated that the frequency sEPSCs in pyramidal neurons were higher in the patients with FCD than in the non-epileptic cortex. This supports previous findings in animal models of CD (Zhu and Roper, 2000). The increased frequency of sEPSCs in pyramidal neurons is associated with a shorter decay time in the non-SOZ of patients with FCD as compared to that in the non-epileptic cortex, which may reflect a high epileptogenicity in the non-SOZ group. However, recent studies have demonstrated that depolarizing GABA activity, caused by imbalanced function of NKCC1 and KCC2, initiates and propagates hypersynchronous neuronal activity into a larger network (Dzhala and Staley, 2021; Ragot et al., 2021). Consistent with the different patterns of NKCC1 and KCC2 during development, GABA initially depolarized immature neurons and controls the early network activity in the developing neocortex (Dzhala et al., 2005; Kirmse et al., 2018). However, Valeeva et al. found a developmental excitatory-to-inhibitory switch in GABA actions on pyramidal neurons from P2–P8 and P9-15 mice *in vitro*. In contrast, mainly inhibitory GABA actions was shown in the immature hippocampus and neocortex *in vivo* (Valeeva et al., 2016). It is that whether traumatic injury might affect results obtained from *in vitro* preparations is currently highly debated (Zilberter, 2016). Further research is required to clarify whether or not this is the case. Human FCD, which has many similarities to the immature cortex, may result in epileptiform synchronization, paradoxically initiated by GABA_A_ activation and GABA_A_R-mediated synaptic transmission changes from inhibitory to excitatory effects caused by altered NKCC1 and KCC2 (D’Antuono et al., 2004; Blauwblomme et al., 2019). In our study, we provided direct neuro-electrophysiological evidence of GABA_A_R-mediated postsynaptic responses, depolarized E_GABA_ in pyramidal neurons at the single-cell level, and corresponding changes in NKCC1 and KCC2 in the SOZ and non-SOZ of patients with FCD exhibiting epilepsy, when compared to those obtained from non-epileptic human temporal neocortex from patients with MTLE. Moreover, we found several differences in GABA activity between the SOZ and non-SOZ groups in the EZ of patients with FCD, highlighting the importance of depolarizing GABA within a seizure network.

### Studying microcircuits in the seizure the onset zone and non-seizure the onset zone may help reveal the mechanism underpinning the generation and spread of macroscopic epileptic networks

In our study, consistent with previous studies, we observed that KCC2 expression was downregulated and internalized in the EZ of patients with FCD, especially in the SOZ (Talos et al., 2012; Blauwblomme et al., 2019). Although decreased expression of KCC2 was noted in both the SOZ and non-SOZ groups, internalized KCC2 was only observed in the SOZ group. Impaired KCC2 regulation may represent a risk factor for the emergence of neuropathology. Pisella et al. (2019) found that heterozygous phosphomimetic variants of KCC2 exhibit altered GABAergic inhibition, increased glutamate/GABA synaptic ratio, and greater susceptibility to seizures. They deduced that dysregulated KCC2 contributed to pathogenesis by controlling the GABAergic developmental sequence *in vivo*, which has been shown to impair neuronal network formation (Pisella et al., 2019). Moreover, normal GABA receptor-mediated inhibition depends on low levels of intracellular Cl^–^, which are modulated by the extrusion of Cl^–^ by KCC2. Thus, the loss of KCC2 function results in the neuronal accumulation of Cl^–^, shifting the direction of ion flow through GABA receptors from hyperpolarizing to depolarizing (Kuchenbuch et al., 2021).

The change of GABA function in the SOZ can strongly affect the microcircuit connection and neuronal network, as well as activate and silence individual neurons. In support of the critical role of KCC2 in postnatal GABAergic inhibition, Kontou et al. (2021) documented rapid mature neuronal apoptosis induced by KCC2 malfunctioning while immature neurons remained mostly intact. This can be explained by the fact that instead of KCC2, NKCC1 is highly expressed in immature neurons and drives Cl^–^ inward. We found that the imbalanced function of NKCC1 and KCC2 in the EZ resulted in depolarization of GABA receptor function and may underpin the hyperexcitability of FCD brain regions. Compared with the control group, SOZ and non-SOZ of patients with FCD showed depolarizing GABA response on pyramidal neurons. Although there were no significant differences in terms of the frequency and intensity of the depolarizing GABA response across the SOZ and non-SOZ groups, fewer neurons with depolarizing GABAergic responses and higher E_GABA_ were shown in the SOZ than in the non-SOZ of FCD patients. The migration of seizures from one side to the other requires the presence of dynamic depolarizing GABA in the network (Kuchenbuch et al., 2021). Therefore, the non-SOZ may represent a subsequent propagation region involved in FCD epileptogenesis. The non-SOZ, with a higher seizure susceptibility, may represent a potential brain region for the initial propagation of epileptic discharges originating in the SOZ. This is consistent with the macroscopic epileptiform discharges observed on EEG recordings that arise in the SOZ and propagate to the non-SOZ. It has also been suggested that more than one potential SOZ, with different thresholds, may exist in a single EZ (Rosenow and Luders, 2001). Although the SOZ with the lowest threshold typically generates seizures, another area in the non-SOZ with the second-lowest threshold would take over to be the new SOZ if the original SOZ has been resected. We speculate that non-SOZ may represent a potential SOZ capable of generating recurrent seizures upon resection of the previous SOZ, which thereby emphasizes the need to remove the entire EZ for seizure-free survival.

### Outlooks and limitations

In our study, we focused on the depolarizing GABA synaptic activity caused by the imbalance of NKCC1 and KCC2 and aimed to build a micro-macro neuronal network to determine the relationship between depolarizing GABA and epileptogenesis in FCD. We observed depolarizing GABAergic signaling among pyramidal neurons in both the SOZ and non-SOZ of the patients with FCD, with a higher number of responsive neurons in the SOZ group. Thus, we speculated that abnormal GABAergic activity might influence epileptogenesis in the SOZ group and seizure susceptibility in the non-SOZ group. The microscopic recordings within areas of macroscopic evaluation and definition, that is, SOZ and non-SOZ, also pointed to the importance and potential of mapping epileptic activities at multiple scales. In addition, given the effects of BUM, the maintenance of chloride homeostasis in neurons could represent an alternative avenue for developing ASMs. Previous studies have reported that BUM exerted significant seizure control effects in adult patients with temporal lobe epilepsy (Eftekhari et al., 2013; Gharaylou et al., 2019). In our study, a BUM application inhibited GABA_A_R-mediated postsynaptic responses in cortical slices of patients with FCD and epilepsy at the individual cell level. However, the use of BUM as a potential new antiepileptic drug warrant further examination. Future studies should examine BUM derivatives as novel ASMs. In addition to NKCC1, an inhibitor of KCC2 should be explored for its effect on depolarizing GABA responses.

This study had several inevitable limitations. First, although we found the specific NKCC1 inhibitor BUM significantly reduced the depolarizing GABAergic response, it is hard to figure out why BUM functions as a result from functional upregulation of NKCC1 or decreased expression of KCC2 in FCD. To solve this issue, we think that the single-channel recording and the specific and available blocker of KCC2 is in urgent need. Second, it remained difficult to obtain SOZ and non-SOZ from the same patient with FCD to avoid excess injury and protect important functional brain areas during surgery. Finally, the size of our cohort was relatively small, and we did not take a deeper look into the differences across variable FCD subtypes and neuronal subgroups. In the future, a larger cohort of patients should be recruited to assess the potentially different GABAergic functions across FCD subtypes and other types of neurons, such as interneurons.

## Data availability statement

The raw data supporting the conclusions of this article will be made available by the authors, without undue reservation.

## Ethics statement

The studies involving human participants were reviewed and approved by the Medical Ethics Committee of Xuanwu Hospital, Capital Medical University. Written informed consent to participate in this study was provided by the participants’ legal guardian/next of kin.

## Author contributions

GZ and XFY: supervision and conceptualization. RL: methodology and writing – original draft. YX, HZ, JW, HLL, and LC: writing – review and editing. DL: visualization. TY, XMY, CX, YP, and LZ: provide clinical patient data, tissue specimens, and pathological examination. HHL: review and edit the manuscript. All authors contributed to the article and approved the submitted version.

## Supplementary material

Supplementary Figure 1 Action potential properties of pyramidal neurons. **(A)** The action potential number of pyramidal neurons (PNs) from control, FCD SOZ, and FCD non-SOZ cortex under a stepped current injection lasting 600 ms. *N* = 16, 19,18 for control and SOZ, non-SOZ groups. **(B)** The first spike latency of PC from control, FCD SOZ, and FCD non-SOZ under a 300 pA injection. *N* = 16, 19, 18 for control and SOZ, non-SOZ groups. Data was shown as mean ± SEM.

Supplementary Figure 2 Expression of NKCC1 in neurons from three groups. Double-label immunostaining for NeuN and NKCC1 demonstrated no obvious differences in expression and sub-cellular localization in neurons from SOZ, non-SOZ and control groups. Arrow indicated neurons with intra-somatic expression of NKCC1. Arrowheads indicated neurons with membrane expression of NKCC1. Scale bar represented 20 μm.
