# Supplementary figures and images for: Imbalance between the function of Na+-K+-2Cl and K+-Cl impairs Cl– homeostasis in human focal cortical dysplasia
Source: Front Mol Neurosci. 2022 Oct 17;15:954167. doi: 10.3389/fnmol.2022.954167 (PMC9621392; doi:10.3389/fnmol.2022.954167)

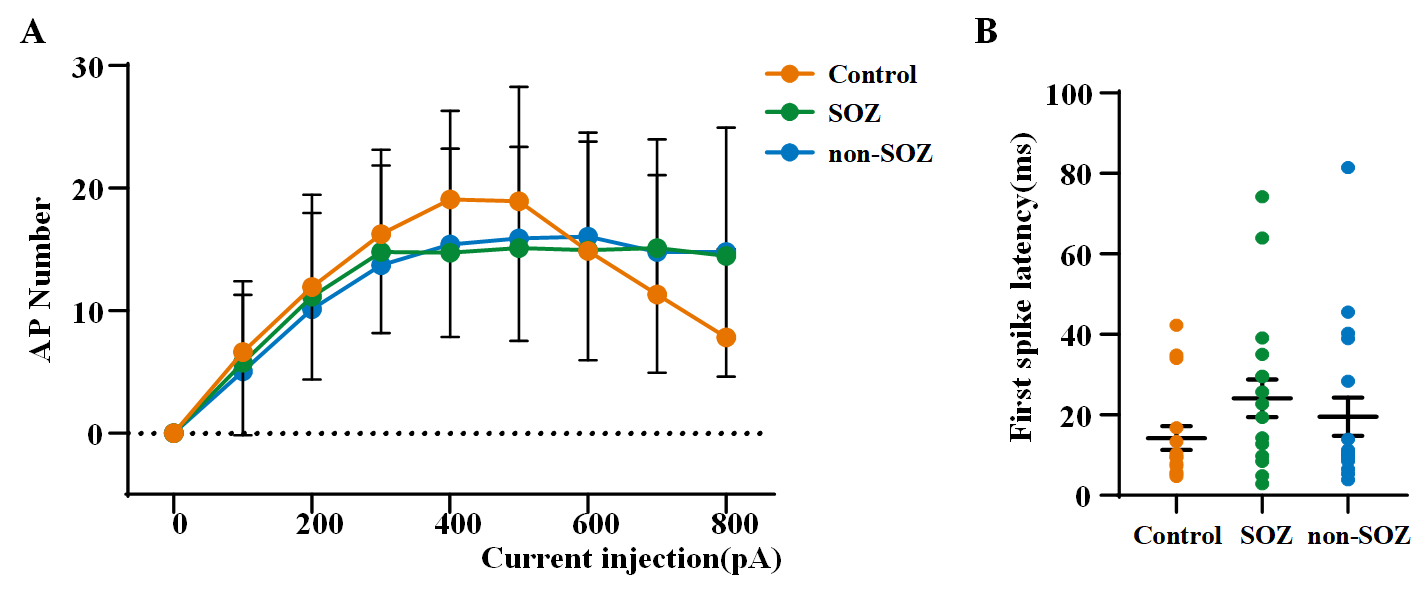

Supplement: Supplementary Figure 1 — Action potential properties of pyramidal neurons. (A) The action potential number of pyramidal neurons (PNs) from control, FCD SOZ, and FCD non-SOZ cortex under a stepped current injection lasting 600 ms. N = 16, 19,18 for control and SOZ, non-SOZ groups. (B) The first spike latency of PC from control, FCD SOZ, and FCD non-SOZ under a 300 pA injection. N = 16, 19, 18 for control and SOZ, non-SOZ groups. Data was shown as mean ± SEM. [file Image_1.TIF]

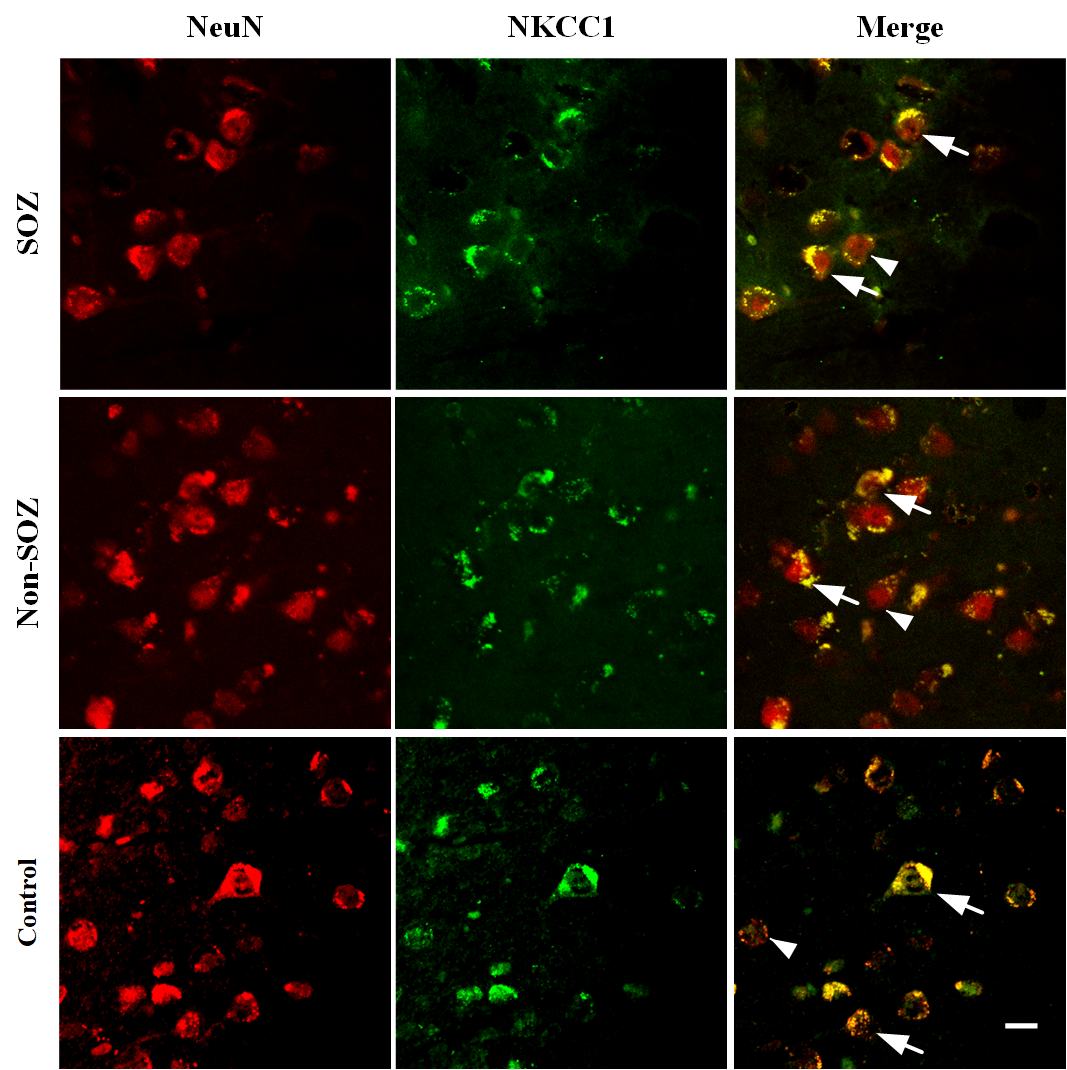

Supplement: Supplementary Figure 2 — Expression of NKCC1 in neurons from three groups. Double-label immunostaining for NeuN and NKCC1 demonstrated no obvious differences in expression and sub-cellular localization in neurons from SOZ, non-SOZ and control groups. Arrow indicated neurons with intra-somatic expression of NKCC1. Arrowheads indicated neurons with membrane expression of NKCC1. Scale bar represented 20 μm. [file Image_2.TIF]
